# Spatiotemporal Co-existence of Two *Mycobacterium ulcerans* Clonal Complexes in the Offin River Valley of Ghana

**DOI:** 10.1371/journal.pntd.0004856

**Published:** 2016-07-19

**Authors:** Araceli Lamelas, Kobina Assan Ampah, Samuel Aboagye, Sarah Kerber, Emelia Danso, Adwoa Asante-Poku, Prince Asare, Julian Parkhill, Simon R. Harris, Gerd Pluschke, Dorothy Yeboah-Manu, Katharina Röltgen

**Affiliations:** 1 Swiss Tropical and Public Health Institute, Basel, Switzerland; 2 University of Basel, Basel, Switzerland; 3 Red de Estudios Moleculares Avanzados, Instituto de Ecologia, A.C., Veracruz, México; 4 Noguchi Memorial Institute for Medical Research, Legon, Ghana; 5 Wellcome Trust Sanger Institute, Cambridge, United Kingdom; Fondation Raoul Follereau, FRANCE

## Abstract

In recent years, comparative genome sequence analysis of African *Mycobacterium ulcerans* strains isolated from Buruli ulcer (BU) lesion specimen has revealed a very limited genetic diversity of closely related isolates and a striking association between genotype and geographical origin of the patients. Here, we compared whole genome sequences of five *M*. *ulcerans* strains isolated in 2004 or 2013 from BU lesions of four residents of the Offin river valley with 48 strains isolated between 2002 and 2005 from BU lesions of individuals residing in the Densu river valley of Ghana. While all *M*. *ulcerans* isolates from the Densu river valley belonged to the same clonal complex, members of two distinct clonal complexes were found in the Offin river valley over space and time. The Offin strains were closely related to genotypes from either the Densu region or from the Asante Akim North district of Ghana. These results point towards an occasional involvement of a mobile reservoir in the transmission of *M*. *ulcerans*, enabling the spread of bacteria across different regions.

## Author Summary

Infection with *Mycobacterium ulcerans* causes the debilitating skin disease Buruli ulcer. Until today, transmission pathways and reservoirs of this emerging pathogen are not well understood. Generally, it is assumed that infection occurs after contact with potential environmental sources of *M*. *ulcerans* through puncture wounds or lacerations or via invertebrate vectors, such as aquatic insects contaminated with the bacteria. Comparative genome analyses of *M*. *ulcerans* strains isolated from patients living in the same BU endemic areas have revealed a close relationship between the genotype detected and the geographical origin, indicating that the reservoir of the pathogen is relatively fixed in space. In the present study, we report the co-circulation of two distinct *M*. *ulcerans* clonal complexes in the same BU endemic area over space and time. Since members of these two clonal complexes were closely related to strains from either the Densu river valley or the Asante Akim North district of Ghana, we conclude that a mobile reservoir of *M*. *ulcerans* may be involved in the occasional spread of the bacteria across different regions.

## Introduction

*Mycobacterium ulcerans* is an emerging pathogen with elusive reservoirs and transmission pathways. It causes the devastating skin disease Buruli ulcer (BU) that mainly affects rural populations in West Africa [1]. *M*. *ulcerans* is a descendant of the fish and occasionally human pathogen *Mycobacterium marinum* [2], from which the new species has evolved through the acquisition of a plasmid encoding the enzymatic machinery for the synthesis of the macrolide toxin mycolactone [3]. From this common ancestor at least three different lineages or ecovars have evolved through genome reduction [4]. Clinical isolates from Africa belong to the classical lineage and differ from each other only in a very limited number of single nucleotide polymorphisms (SNPs) [4, 5], indicative for a highly clonal recent expansion of the pathogen in Africa. BU is characterized by a focal distribution of cases within endemic countries. Previous studies have revealed a strong association between genotype and the geographic origin of strains [4, 6, 7], speaking for the development of local clonal *M*. *ulcerans* complexes following the introduction of this pathogen into a new area. The limited genomic diversity found within these local clonal complexes is however sufficient for studies on the distribution of variants at a micro-epidemiological level [8]. Since human-to-human transmission seems to be rare, findings point towards infection from a relatively localized environmental reservoir of the pathogen. In view of the association of BU outbreaks with stagnant and slow-flowing water bodies, a reservoir in the aquatic ecosystem is considered likely [9]. While in several African endemic areas a single unique clonal complex has been identified [4, 6–8], a recent comparative whole genome sequencing study of isolates from residents of the Asante Akim North district of Ghana showed for the first time the concurrent presence of two distinct clonal complexes within one BU endemic area [10].

Within the framework of a comprehensive genome analysis of clinical *M*. *ulcerans* isolates from Ghana, we analyzed genomes of a limited number of strains from the Offin river valley and equally observed a co-existence of two clonal complexes.

## Methods

### Ethics statement

Ethical approval for the study was obtained from the institutional review board of the Noguchi Memorial Institute for Medical Research (Federal-wide Assurance number FWA00001824). Written informed consent was provided by all study participants.

### Study area and *M*. *ulcerans* isolates

In an exhaustive active BU case search conducted in 2013 and a subsequent continuous monitoring of cases over a 17-months period in 13 randomly selected communities located in the historically highly BU endemic Offin river valley, an unexpectedly low prevalence of BU was revealed with only 11 laboratory-confirmed cases identified [11]. Two *M*. *ulcerans* strains that could be isolated from two of the 11 patients, as well as three *M*. *ulcerans* strains isolated in 2004 from lesions of two BU patients residing in the valley were analyzed in this study (Table 1). In addition, we included 48 *M*. *ulcerans* isolates from patients residing in BU endemic areas located in the Densu river valley of Ghana, which were part of a previous SNP typing study [8]. All *M*. *ulcerans* strains were subjected to whole genome sequencing.

**Table 1:** *M*. *ulcerans* Offin isolates sequenced in this study.

Isolate ID	Year of isolation	Village of residence*	Genotype	Average sequence coverage
**NM022B**	2004	Atuntuma	Agogo-1	101.54
**NM022D**	2004	Atuntuma	Agogo-1	88.304
**NM031**	2004	Subin	Densu	91.743
**NM972**	2013	Treposo	Agogo-1	90.481
**NM997**	2013	Ntobroso	Densu	94.425

*see Fig 4 for location along the Offin river

### DNA extraction and whole genome analysis

Genomic DNA was extracted from *M*. *ulcerans* cultures by phenol-chlorophorm extraction and ethanol precipitation as described previously [12]. Multiplexed genomic DNA libraries were prepared and sequenced on an Illumina HiSeq 2000 on 75-bp paired-end runs [13]. Illumina reads were aligned to the complete reference genome of *M*. *ulcerans* strain Agy99 (GenBank accession number CP000325.1) with an insert size between 50 and 400 bp using BWA version 0.7.10. SNPs were identified using SAMtools [14] as described [15] and were filtered for a minimum mapping quality of 30 and a quality cutoff of 75%. SNPs called in repetitive regions of the *M*. *ulcerans* reference genome (737,280 bp) were excluded from the analysis and only the SNPs mapped in the core genome (4,894,326 bp) were used to construct the phylogenetic trees.

### Phylogenetic analyses

Maximum-likelihood phylogenetic analysis was performed using RAxML [16] on the alignment of identified SNPs from across the Ghanaian genomes sequenced here, together with genomes sequenced in previous studies [4, 10, 17]. Additional strains isolated from BU patients from other regions of Ghana and from Benin and Australia were included in the analysis to provide a comprehensive genetic context for the analysis of genetic diversity among the Offin and Densu isolates.

## Results/Discussion

**Fig 1:**
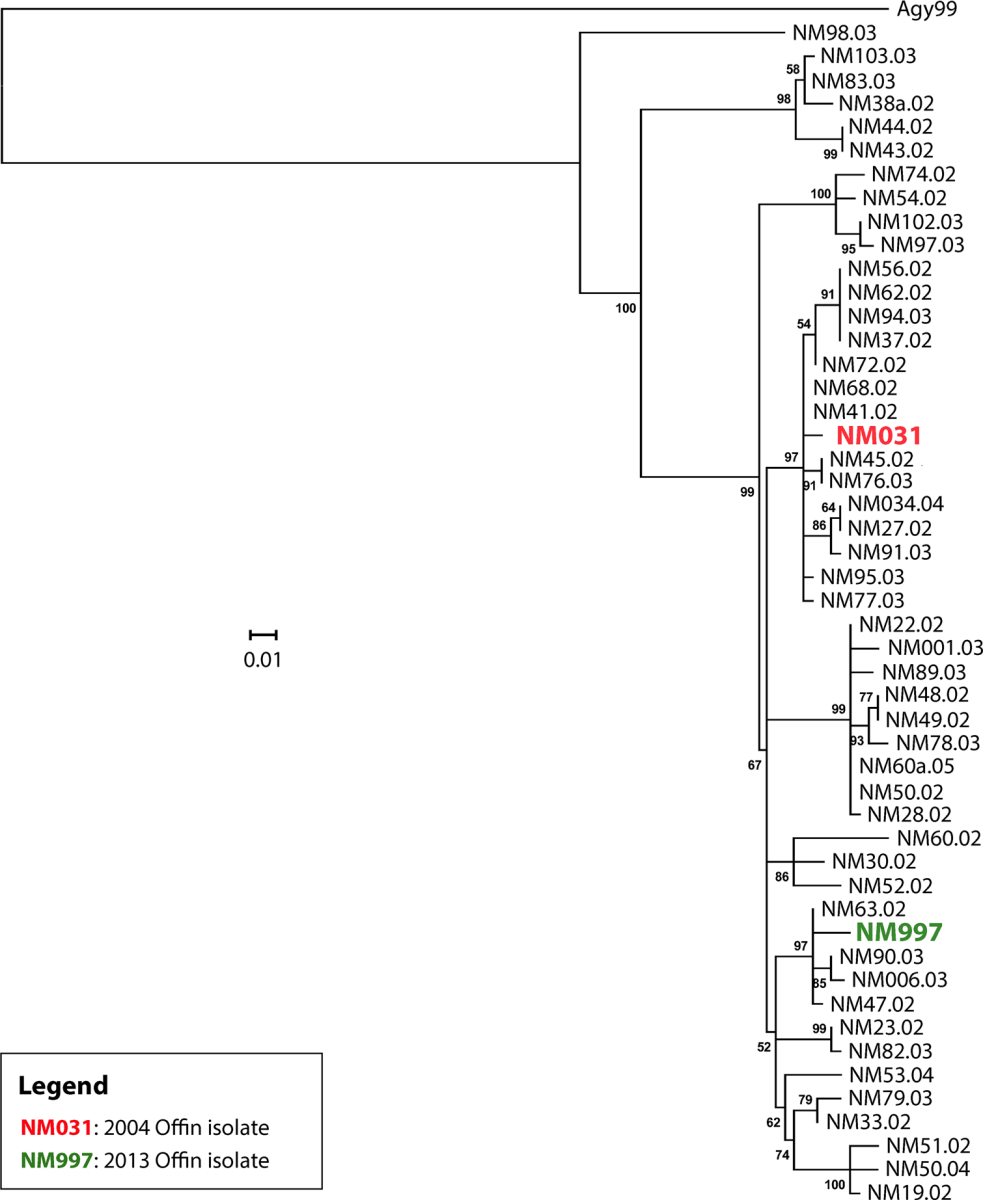
Phylogenetic reconstruction of *M*. *ulcerans* isolates belonging to the clonal complex circulating in the Densu river valley of Ghana. Maximum-likelihood phylogenetic tree based on 292 variable nucleotide positions across *M*. *ulcerans* isolates produced by RAxML. A total of 48 *M*. *ulcerans* isolates from the Densu river valley and two isolates from the Offin river valley were shown to belong to the same clonal complex that has diversified substantially over time. The tree was rooted using *M*. *ulcerans* strain Agy99 as an out-group. Bootstrap values are shown along the branches. 0.01 = scale for genetic distance.

**Fig 2:**
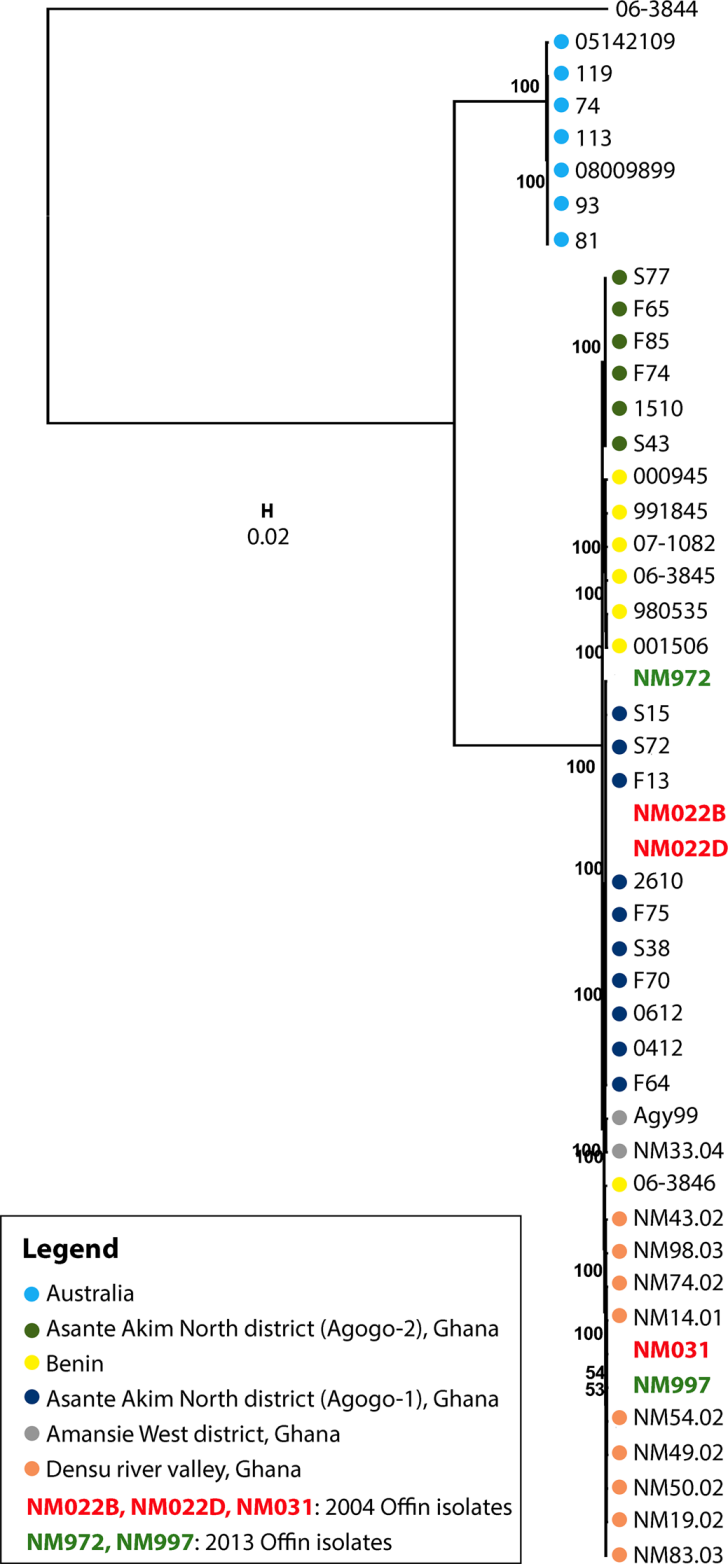
Phylogenetic reconstruction of *M*. *ulcerans* isolates belonging to the classical lineage (Australian and African strains). Maximum-likelihood phylogenetic tree based on 11,194 variable nucleotide positions across *M*. *ulcerans* isolates by RAxML. *M*. *ulcerans* isolates from the Offin and Densu river (one representative each of the previously identified SNP haplotypes [8]) valleys were placed in a broader genomic context. The tree was rooted using *M*. *ulcerans* strain Mu 06–3844 (isolate from a fish farm in Belgium) as an out-group. Bootstrap values are shown along the branches. 0.02 = scale for genetic distance.

**Fig 3:**
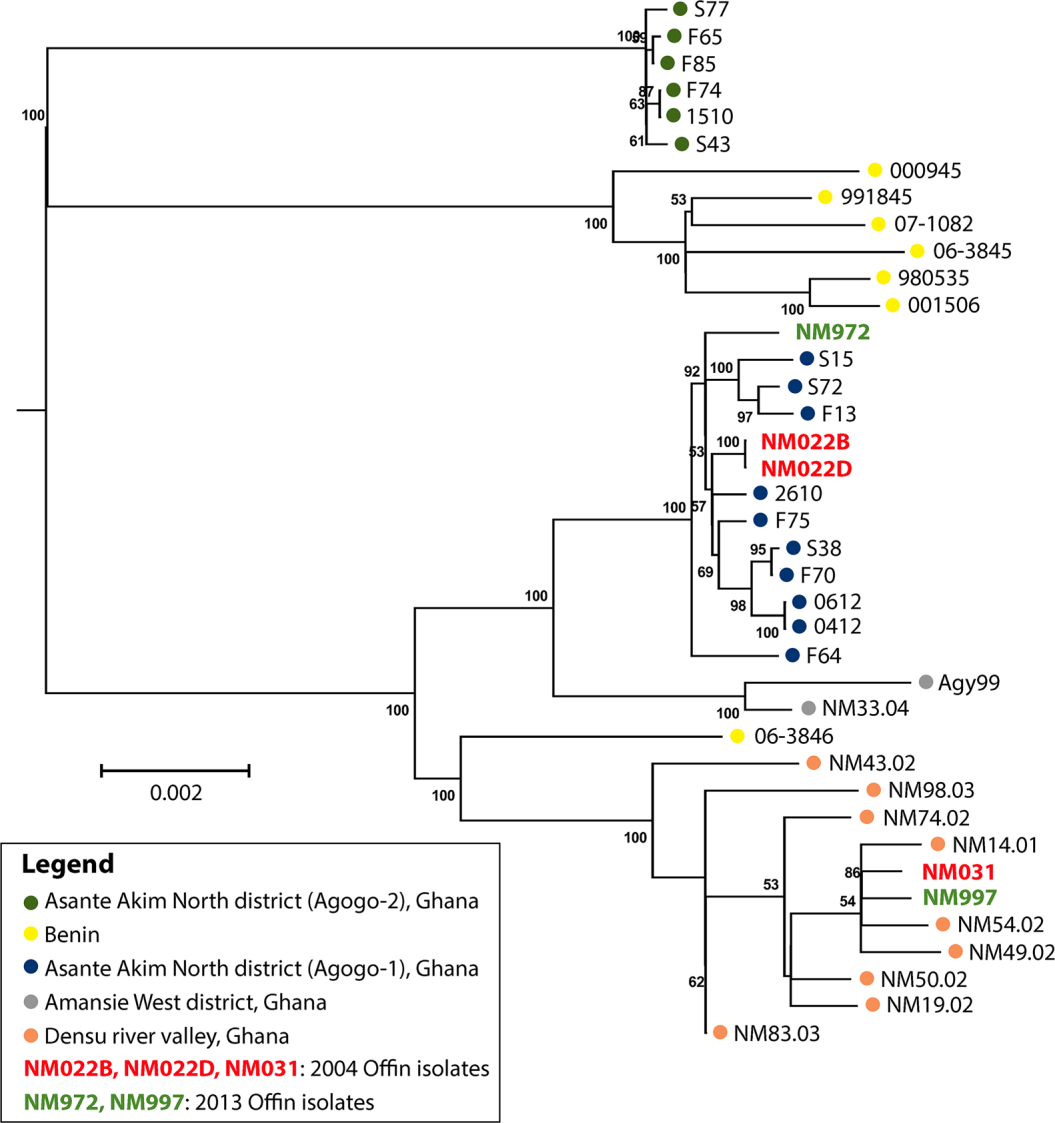
Phylogenetic reconstruction of closely related *M*. *ulcerans* isolates from different BU endemic areas of Ghana and from Benin. Maximum-likelihood phylogenetic tree based on 776 variable nucleotide positions showing relations between the strains at a higher resolution than in Fig 2. Bootstrap values are shown along the branches. 0.002 = scale for genetic distance.

**Fig 4:**
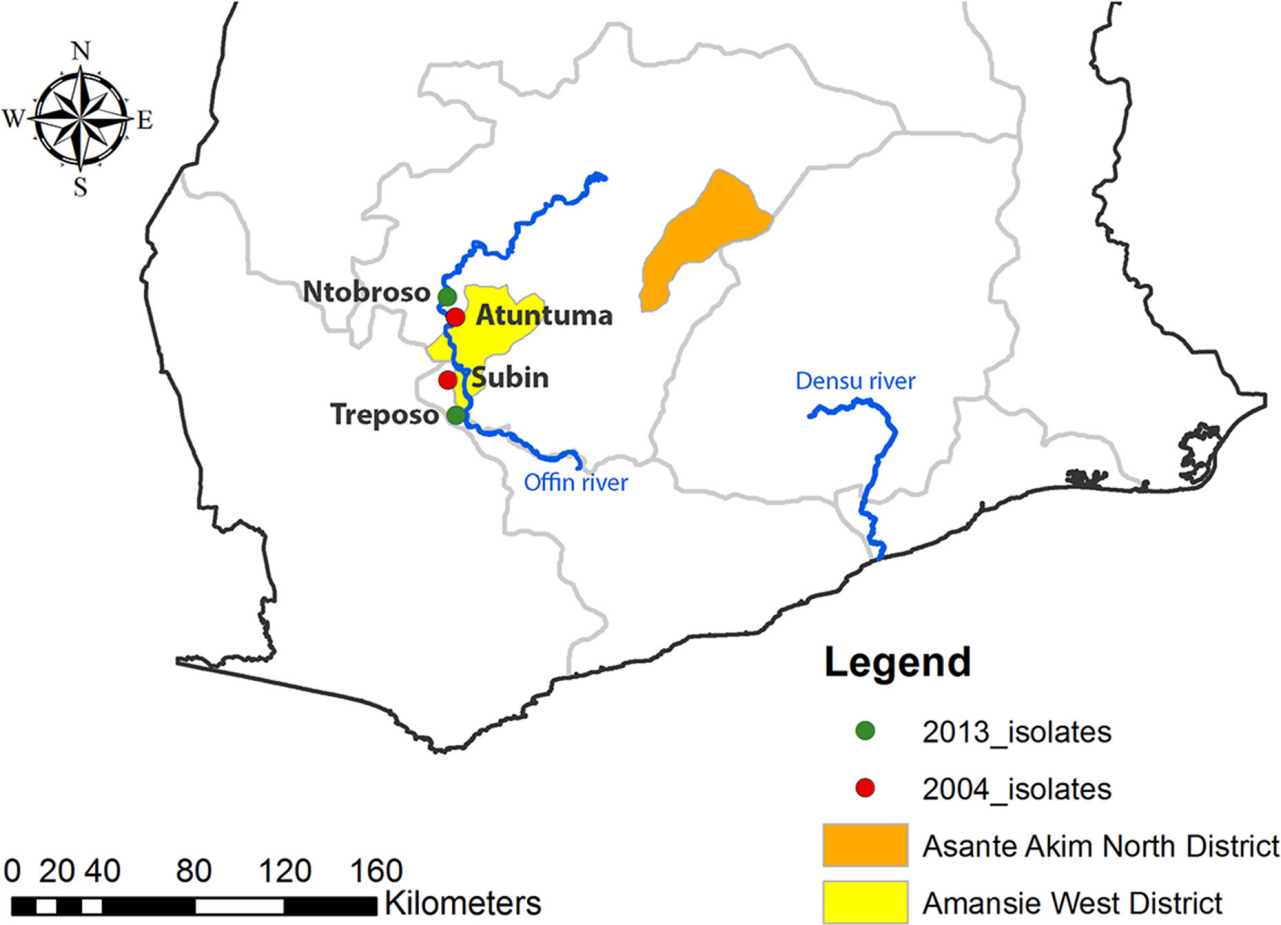
Geographical distribution of *M*. *ulcerans* isolates from the Offin river valley. Map of the Offin river basin, depicting the residences of BU patients from whom the strains have been isolated in 2004 (red) and 2013 (green). The background map was created using the *ArcMap* program in *ArcGIS* v.10.0 software.

Phylogenetic analysis demonstrated the expansion of a single clonal complex in the Densu river valley (Fig 1). This complex has diversified substantially, but still forms a separate cluster, distinct from other African local clonal complexes (Figs 2 and 3). When compared to the second branch of the classical lineage of *M*. *ulcerans*—isolates from Australia—it is evident, that all African isolates are genomically extremely closely related (Fig 2). In contrast to the observation of a single clonal complex in the Densu river valley, our analysis revealed for the Offin river valley the presence of members of two distinct clonal complexes (Fig 3). Two Offin isolates (NM031 and NM997) were closely related to isolates from the Densu river valley of Ghana (Figs 1 and 3). The other three Offin isolates (NM022B, NM022D and NM972)–separated from the two Densu-like Offin strains by 29 SNPs–clustered with strains (belonging to a clonal complex designated Agogo-1; [10]) from the Asante Akim North district in the Ashanti region of Ghana (Fig 3). Intra-genotype average diversity was low with 12 and 17 identified SNPs among Densu-like and Agogo-1-like Offin isolates, respectively. Not a single SNP difference was found between the genomes of two strains (NM022B and NM022D) isolated from two different lesions of the same patient. In a next step, we combined the phylogenetic analysis of the Offin isolates with information on the residence of the patients within the river valley and the year of strain isolation (Fig 4). In both 2004 and 2013 one member each of the two clonal complexes, isolated from BU patients resident in different communities was found. Due to the limited number of isolates and missing details on the travel history of the BU patients from which these strains have been isolated, no firm conclusions could be drawn concerning the apparent lack of geographical clustering. However, the data revealed a co-circulation of two distinct *M*. *ulcerans* clonal complexes in the Offin river valley over space and time.

In contrast to the remarkably strong link between genotype and geographical origin of clinical *M*. *ulcerans* isolates reported in previous genotyping studies conducted in African BU endemic foci [6–8], two distinct *M*. *ulcerans* clonal complexes were recently found to co-exist in the Asante Akim North district of Ghana among strains isolated within the short time frame of two years and an area of only 30km^2^ [10]. It was concluded that *M*. *ulcerans* genotypes might be spread across larger areas, suggesting the presence of a rather mobile reservoir of infection in addition to the postulated more focalized aquatic niche environment typically associated with the pathogen [9]. In this context, recent data indicated that *M*. *ulcerans* is able to persist for several months in underwater decaying organic matter [18], possibly as a commensal in protective aquatic host environments [19–21]. While the specific factors favoring the persistence of *M*. *ulcerans* in the environment and its transmission are yet to be explored, a complex interplay between environmental factors as well as biotic and abiotic drivers is assumed [22, 23]. Reductive genome evolution of *M*. *ulcerans* speaks for niche adaptation [17].

In the present study we revealed the co-existence of both Densu-like and Agogo-1-like *M*. *ulcerans* genotypes in communities along the Offin river at two time points separated by ten years. Our data thus show that co-existence of clonal complexes in one BU endemic area may prevail over longer time periods. A mobile mammalian host, allowing the bacteria to replicate and to be shed to the environment, that way forming a reservoir [18] from which humans may be infected by unknown mechanisms, could be the missing link explaining the spread of *M*. *ulcerans* from an established BU endemic region to a new area. However, as demonstrated here by the presence of only a single clonal complex in the Densu river valley, the exchange of genetic *M*. *ulcerans* variants between BU endemic areas appears to be an extremely rare event.

While in Australia possums have been identified as a host for *M*. *ulcerans* [24], another line of evidence points to the involvement of humans with chronic ulcerative BU lesions in the spread of the bacteria in African BU environments. Extensive whole-genome sequencing studies are required to further unravel the evolutionary history and population structure of *M*. *ulcerans* in Africa.
